# Natural history observations in moderate aortic stenosis

**DOI:** 10.1186/s12872-021-01901-1

**Published:** 2021-02-19

**Authors:** Yu Du, Mario Gössl, Santiago Garcia, Maurice Enriquez-Sarano, Joao L. Cavalcante, Richard Bae, Go Hashimoto, Miho Fukui, Bernardo Lopes, Aisha Ahmed, Christian Schmidt, Larissa Stanberry, Ross Garberich, Steven M. Bradley, Robert Steffen, Paul Sorajja

**Affiliations:** 1 Roger L. and Lynn C. Headrick Chair, Valve Science Center, Minneapolis Heart Institute Foundation, Abbott Northwestern Hospital, Minneapolis, MN 55407 USA; 2 Department of Cardiology, Beijing Anzhen Hospital, Capital Medical University, Beijing, 100029 China

**Keywords:** Moderate, Aortic stenosis, Outcomes, Survival, Aortic valve replacement

## Abstract

**Background:**

The natural history of patients with moderate aortic stenosis (AS) is poorly understood. We aimed to determine the long-term outcomes of patients with moderate AS.

**Methods:**

We examined patients with moderate AS defined by echocardiography in our healthcare system, and performed survival analyses for occurrence of death, heart failure (HF) hospitalization, and progression of AS, with accounting for symptoms, left ventricular dysfunction, and comorbidities.

**Results:**

We examined 729 patients with moderate AS (median age, 76 years; 59.9 % men) with a median follow-up of 5.0 years (interquartile range: 2.0 to 8.1 years). The 5-year overall survival was 52.3 % (95 % confidence interval [CI]: 48.6 % to 56.0 %) and survival free of death or HF hospitalization was 43.2 % (95 % CI: 39.5 % to 46.9 %). Worse New York Heart Association (NYHA) functional class was associated with poor long-term survival, with mortality rates ranging from 7.9 % (95 % CI: 6.6–9.2 %) to 25.2 % (95 % CI: 20.2–30.3 %) per year. Among patients with minimal or no symptoms, no futility markers, and preserved left ventricular function, 5-year overall survival was 71.9 % (95 % CI: 66.4–77.4 %) and survival free of death or HF hospitalization was 61.4 % (95 % CI: 55.5–67.3 %). Risk factors associated with adverse events were age, NYHA class, low ejection fraction and high aortic valve velocity (all *p* < 0.05).

**Conclusions:**

Patients with moderate AS are at significant risk of death. Our findings highlight the need for more study into appropriate therapeutic interventions to improve the prognosis of these patients.

## Background

Aortic stenosis (AS) is one of the most common valvular diseases, with a growing number of patients due to population aging. Among those over 65 years of age, the prevalence of moderate or severe AS is 2 to 4 %, with 1.5 to 3 million afflicted patients in the U.S. alone [1]. Thresholds for intervention have been traditionally defined by assessment of symptoms and echocardiographic parameters of stenosis severity [2, 3]. To date, the major focus in the management of AS has been on the identification of those with symptomatic severe stenosis, in whom aortic valve replacement (AVR) is well established as a life-saving therapy [2, 3]. Nevertheless, AS, even when defined as moderate in severity, may impart hemodynamic effects that could burden patients and potentially be associated with heart failure (HF) and impairment in long-term survival [4–9]. Such observations have implications for treatment thresholds for patients with AS, but remain limited due to confounding factors such as symptoms, morbidities, and concomitant left ventricular (LV) dysfunction [4–9]. Accordingly, we undertook this real-world, longitudinal study of a large cohort of patients with moderate AS in order to better define the natural history of moderate AS.

## Methods

### Study population

The Allina Health System is a private, nonprofit healthcare system that provides care for patients from Minnesota and western Wisconsin, comprised of 3 tertiary and 9 rural hospitals, and more than 90 outpatient clinics. All patients seen between January 2010 and March 2012 in the Allina Health System were considered for enrollment in the present investigation. We examined the initial two-dimensional and Doppler echocardiogram (index echocardiogram) of each patient seen during this time period, and enrolled those with a diagnosis of moderate AS, as defined by the presence of one of the following criteria: (1) peak aortic velocity of 3.0 to 4.0 m/s; (2) mean aortic gradient of 20 to 40 mmHg; (3) aortic valve area (AVA) of 1.0 to 1.5 cm^2^; (4) indexed AVA of 0.60 to 0.85 cm^2^/ m^2^; or (5) a dimensionless index (DI) of 0.25 to 0.50 [10]. Exclusion criteria for the study were: (1) age < 18 years; (2) prior AVR or aortic valve repair; (3) severe mitral, tricuspid, or pulmonary valve disease; (4) severe aortic regurgitation or evidence of supra- or sub-aortic stenosis; and (5) hemodynamic findings consistent with severe AS (i.e., peak aortic velocity ≥4 m/s, mean aortic gradient ≥40 mmHg, or AVA < 1.0 cm^2^). The study was approved by the Allina Institutional Review Board and was performed in accordance with the Declaration of Helsinki.

## Data collection and definitions

Each electronic medical record was reviewed for patient demographics, cardiac symptoms, and morbidities present at the time of the index echocardiogram, and subsequent clinical outcomes. The index echocardiogram was the first echocardiogram during the study period that identified moderate AS patients without exclusion criteria for enrollment. AVA was calculated using the continuity equation. DI was calculated as the ratio of the LV outflow tract velocity time integral (VTI) and to the aortic valve VTI [11]. LV mass index was calculated using the Devereux formula [12]. Patients were classified into New York Heart Association (NYHA) functional class on the basis of the presence of dyspnea. Potential futility markers were defined as: (1) oxygen-dependent severe lung disease; (2) liver disease with model for end-stage liver disease score ≥ 12; (3) end-stage renal disease on dialysis; (4) excessive frailty; (5) severe dementia; or (6) malignancy with life expectancy < 1 year [13–15].

Clinical outcomes in follow-up evaluations were obtained by review of medical records or telephone interview. The primary clinical outcome of interest in the study was all-cause mortality. Other clinical outcomes were occurrence of HF hospitalization, the combined endpoint of death or HF hospitalization, myocardial infarction (MI), stroke, progression of AS to the severe range, and occurrence of transcatheter or surgical AVR. Standard definitions of HF hospitalization, MI, and stroke were utilized [16, 17].

### Data analysis

Three patient groups were defined according to baseline NYHA functional class (I, II, or III/IV). Continuous data were summarized by medians and interquartile ranges (IQR), and compared using a Kruskal-Wallis H test. Categorical variables were summarized by counts (%) and compared using either a Chi-squared test or a Fisher’s exact test as appropriate. For each endpoint, incidence rates per 100 person-years of follow-up and their 95 % confidence intervals (CI) were calculated using a Poisson distribution. Kaplan-Meier survival curves were estimated for each primary endpoint and compared between different NYHA functional classes using a log-rank test, with and without censoring for occurrence of AVR, and with and without exclusion of patients with potential futility markers or LV ejection fraction < 50 %. Further, a one-sample log-rank test was also used to compare the survival free of death for the select patient subgroups with that of the total age- and gender-matched Minnesota population based on the 2013 life tables. Individual expected survival estimates were computed and used to estimate the incident risks of all-cause mortality with a Poisson model adjusted for a sex-specific baseline mortality due to age.

Multivariate Cox proportional hazard models were used to estimate the association between the risk of adverse events and underlying clinical and demographic factors with AVR modeled as a time-dependent variable. All models were stratified by gender, adjusted for age and body surface area, and included NYHA functional class; the body surface area was included to account for the effect of body size on cardiovascular parameters. Other candidate explanatory variables collected at index echocardiogram including continuous measurements of blood pressure, blood pressure medication prescription, mean aortic gradient, peak aortic velocity, LV ejection fraction, body mass index, stroke volume index; binary indicators of moderate or severe mitral regurgitation, moderate or severe tricuspid regurgitation, moderate or severe right ventricular systolic dysfunction, hypertension, diabetes, current smoking status, coronary artery disease, prior MI, prior percutaneous coronary intervention, prior coronary artery bypass grafting, prior stroke or transient ischemic attack, chronic lung disease, peripheral artery disease, dialysis, anemia, malignancy, permanent pacemaker, implanted defibrillator. Also included were futility markers of oxygen-dependent severe lung disease, end-stage renal disease, and excessive frailty; since only two patients in the study had end-stage liver disease and only four had severe dementia, these markers were not included separately but aggregated into broader binary indicators of dementia and liver disease; malignancy was included as a three-level factor: none, malignancy with expected survival of more and of less than one year. A final model was constructed using a stepwise forward selection and backward elimination algorithm with a generalized Akaike information criterion. The estimated associations are reported together with their 95 % CI and p-values. The variable selection and analyses were repeated for patients without futility markers. Data analysis was conducted using SPSS 22 (IBM, Armonk, New York) and R v4.0 in RStudio v1.3 environment (RStudio PBC).

## Results

## Patient characteristics

Overall, 729 patients with moderate AS (median age, 76 years [IQR: 67 to 84 years]; 59.9 % men) were enrolled in the study (Table 1, Additional file 1: Figure 1). The vast majority (93.1 %) were white, and with a history of hypertension (80.2 %) or dyslipidemia (71.3 %). Coronary artery disease was present in 365 patients (50.1 %) with 197 patients (27.0 %) having prior MI. Overall, 423 patients (58.0 %) had symptoms of dyspnea that were more than mild (i.e., NYHA functional class II or worse). The prevalence of morbidities increased with worse NYHA class, and potential futility markers were present in 90 patients (or 12.3 %) in the overall study population.

**Table 1:** Baseline patient characteristics

	All patients N = 729	NYHA I N = 306	NYHA II N = 309	NYHA III or IV N = 114	*p*
Age (yrs)	76 (67, 84)	74 (66, 83)	77 (68, 85)	77 (69, 84)	0.107
Women	292 (40.1)	139 (45.4)	116 (37.5)	37 (32.5)	0.027
Caucasian	679 (93.1)	287 (93.8)	289 (93.5)	103 (90.4)	0.435
Body mass index (kg/m^2^)	27.7 (24.5, 32.2)	28.0 (24.8, 32.3)	27.9 (24.5, 32.2)	26.8 (23.7, 31.9)	0.290
Hypertension	585 (80.2)	236 (77.1)	251 (81.2)	98 (86.0)	0.110
Diabetes	236 (32.4)	81 (26.5)	112 (36.2)	43 (37.7)	0.014
Dyslipidemia	520 (71.3)	201 (65.7)	240 (77.7)	79 (69.3)	0.004
Current smoking	68 (9.3)	28 (9.2)	29 (9.4)	11 (9.6)	0.987
Coronary artery disease	365 (50.1)	101 (33.0)	196 (63.4)	68 (59.6)	< 0.001
Prior myocardial infarction	197 (27.0)	32 (10.5)	114 (36.9)	51 (44.7)	< 0.001
Prior PCI	216 (29.6)	61 (19.9)	118 (38.2)	37 (32.5)	< 0.001
Prior CABG	138 (18.9)	30 (9.8)	79 (25.6)	29 (25.4)	< 0.001
Atrial fibrillation or flutter	286 (39.2)	91 (29.7)	134 (43.4)	61 (53.5)	< 0.001
Stroke or TIA	128 (17.6)	48 (15.7)	52 (16.8)	28 (24.6)	0.095
Chronic lung disease	146 (20.0)	40 (13.1)	74 (23.9)	32 (28.1)	< 0.001
O_2_-dependent	31 (4.3)	8 (2.6)	17 (5.5)	6 (5.3)	0.163
Peripheral artery disease	121 (16.6)	37 (12.1)	63 (20.4)	21 (18.4)	0.018
CKD stage ≥ III	207 (28.6)	45 (14.8)	109 (35.5)	53 (46.9)	< 0.001
Dialysis	28 (3.8)	4 (1.3)	13(4.2)	11 (9.6)	0.001
Anemia	350 (48.0)	120 (39.2)	152 (49.2)	78 (68.4)	< 0.001
Permanent pacemaker	101 (13.9)	18 (5.9)	60 (19.4)	23 (20.2)	< 0.001
Implanted defibrillator	70 (9.6)	11 (3.6)	32 (10.4)	27 (23.7)	< 0.001
Severe liver cirrhosis	2 (0.3)	0 (0)	1 (0.3)	1 (0.9)	0.288
Severe dementia	4 (0.5)	0 (0)	1 (0.3)	3 (2.6)	0.013
Life-threatening malignancy	18 (2.5)	9 (2.9)	7 (2.3)	2 (1.8)	0.809
Excessive frailty	25 (3.4)	4 (1.3)	13 (4.2)	8 (7.0)	0.006
Medical therapy					
Anti-platelet	527 (72.3)	203 (66.3)	233 (75.4)	91 (79.8)	0.006
Warfarin or NOAC	203 (27.8)	61 (19.9)	97 (31.4)	45 (39.5)	< 0.001
ACE-i or ARB	404 (55.4)	141 (46.1)	193 (62.5)	70 (61.4)	< 0.001
Beta-blocker	511 (70.1)	172 (56.2)	239 (77.3)	100 (87.7)	< 0.001
Nitrates	83 (11.4)	19 (6.2)	38 (12.3)	26 (22.8)	< 0.001
Diuretic	379 (52.0)	118 (38.6)	176 (57.0)	85 (74.6)	< 0.001
Aldosterone antagonist	54 (7.4)	11 (3.6)	22 (7.1)	21 (18.4)	< 0.001

Data are reported as median (IQR) or no. (%). ACE-i, angiotensin converting enzyme inhibitor; ARB, angiotensin receptor antagonist; CABG, coronary artery bypass grafting; CKD, chronic kidney disease; PCI, percutaneous coronary intervention; NOAC, novel oral anticoagulant; NYHA, New York Heart Association; TIA, transient ischemic attack

Overall, the median AVA was 1.51 cm^2^ (IQR: 1.34 to 1.73 cm^2^) with a median aortic valve gradient of 9.1 mmHg (IQR: 5.9 to 14.0 mmHg) (Table 2). The LV ejection fraction was preserved in the majority of patients (71.6 %) with a median value of 58 % (IQR: 45 % to 63 %). Worse NYHA class was associated with lower aortic valve velocity, mean aortic gradient, LV ejection fraction, and stroke volume index (all *p* < 0.001). Higher AVA, larger LV end-diastolic diameter, and more mitral and tricuspid regurgitation also were more common in those with worse NYHA functional class (all *p* < 0.01).

**Table 2:** Echocardiographic data

	All patients N = 729	NYHA I N = 306	NYHA II N = 309	NYHA III or IV N = 114	*p*
Peak aortic velocity (m/s)	2.1 (1.7, 2.6)	2.3 (1.9, 2.8)	2.0 (1.6, 2.3)	1.8 (1.5, 2.2)	< 0.001
Mean aortic gradient (mmHg)	9.1 (5.9, 14.0)	11.8 (7.6, 16.5)	8.0 (5.3, 11.8)	6.4 (4.4, 11.1)	< 0.001
Aortic valve area (cm^2^)	1.51 (1.34, 1.73)	1.48 (1.30, 1.70)	1.55 (1.37, 1.74)	1.57 (1.38, 1.75)	0.004
Aortic valve area index (cm^2^/ m^2^)	0.79 (0.69, 0.89)	0.76 (0.68, 0.86)	0.80 (0.71, 0.91)	0.79 (0.70, 0.90)	0.002
Dimensionless index	0.44 (0.39, 0.47)	0.43 (0.38, 0.47)	0.44 (0.40, 0.47)	0.44 (0.38, 0.48)	0.196
Stroke volume index (ml/m^2^)	33.8 (25.6, 41.2)	37.1 (30.0, 44.3)	31.4 (25.1, 39.5)	28.2 (23.0, 36.0)	< 0.001
Stroke volume index <35 ml/m^2^	357 (54.3)	104 (38.5)	178 (62.7)	75 (72.1)	< 0.001
Left ventricular EDD (cm)	4.8 (4.2, 5.4)	4.5 (4.0, 5.1)	4.8 (4.2, 5.4)	5.4 (4.9, 6.0)	< 0.001
Left ventricular mass index (g/m^2^)	112 (91, 137)	101 (83, 124)	116 (96, 139)	132 (108, 161)	< 0.001
Left ventricular ejection fraction (%)	58 (45, 63)	60 (58, 65)	55 (40, 63)	38 (23, 58)	< 0.001
Left ventricular ejection fraction <50 %	207 (28.4)	23 (7.5)	112 (36.2)	72 (63.2)	< 0.001
E/e’	12.0 (9.0, 16.0)	11.0 (8.5, 15.0)	12.0 (9.0, 17.0)	13.0 (10.9, 18.0)	0.006
Left atrial volume index (ml/ m^2^)	37 (29, 49)	34 (27, 46)	36 (29, 50)	45 (34, 56)	< 0.001
RV systolic pressure (mmHg)	35 (28, 44)	33 (27, 40)	36 (28, 46)	43 (36, 51)	< 0.001
≥ moderate MR	61 (8.4)	10 (3.3)	34 (11.1)	17 (14.9)	< 0.001
≥ moderate TR	96 (13.5)	31 (10.4)	38 (12.7)	27 (24.3)	0.001
≥ moderate RV dysfunction	33 (4.7)	2 (0.7)	13 (4.3)	18 (16.8)	< 0.001

Data are reported as median (IQR) or no. (%). EDD, end-diastolic dimension; LV, left ventricle; MR, mitral regurgitation; NYHA, New York Heart Association; RV, right ventricular; TR, tricuspid regurgitation

## Clinical outcomes

**Fig. 1:**
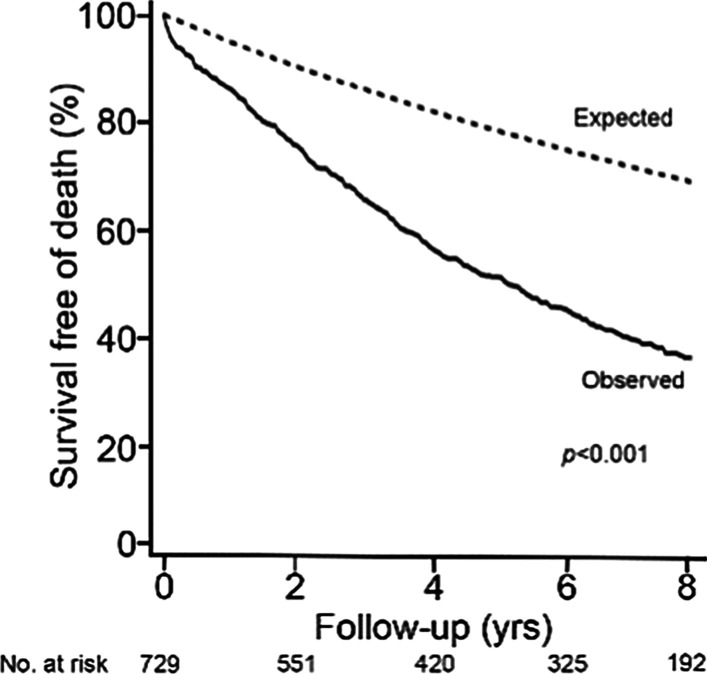
Survival of patients with moderate aortic stenosis**.** Observed survival free of all-cause mortality (solid) for the entire study cohort in comparison to the expected survival, based on the age- and sex-matched total Minnesota population (dashed), is shown

Clinical follow-up was complete in all patients with a median time of 5.0 years (IQR: 2.0 to 8.1 years), and any death occurred in 453 patients (or 62.1 %) with an estimated survival time of 5.5 years (95 % CI: 4.8 to 6.2 years). Overall, 5-year survival was 52.3 % (95 % CI: 48.6 % to 56.0 %), with an incidence of 12.6 (95 % CI: 11.4 to 13.7) per 100 person-year (Fig. 1; Table 3). In the entire study group, HF hospitalization occurred in 211 patients (or 28.9 %), with an incidence of 6.7 (95 % CI: 5.8 to 7.6) per 100 person-year (Table 3). For the combined endpoint of death or HF hospitalization, the 5-year survivorship was 43.2 % (95 % CI: 39.5 % to 46.9 %), with an incidence of 16.3 (95 % CI: 14.9 to 17.7) per 100 person-year (Table 3).

**Table 3:** Major adverse clinical events

All patients	Overall N = 729	NYHA I N = 306	NYHA II N = 309	NYHA III or IV N = 114	*P*
Any death	12.6 (11.4, 13.7)	7.9 (6.6, 9.2)	15.1 (13.1, 17.1)	25.2 (20.2, 30.3)	< 0.001
Heart failure hospitalization	6.7 (5.8, 7.6)	4.5 (3.4, 5.5)	7.9 (6.3, 9.4)	13.9 (9.8, 18.1)	0.014
Any death or heart failure hospitalization	16.3 (14.9, 17.7)	10.7 (9.2, 12.3)	19.5 (17.0, 22.0)	33.4 (26.9, 39.8)	< 0.001
Progression to severe aortic stenosis*	3.9 (3.1, 4.7)	4.3 (3.1, 5.5)	3.5 (2.3, 4.7)	3.2 (0.8, 5.5)	0.014
AVR	2.5 (1.9, 3.0)	3.0 (2.2, 3.9)	1.9 (1.2, 2.7)	1.9 (0.5, 3.4)	0.001
Any death, heart failure hospitalization, or AVR	18.4 (16.9, 20.0)	13.1 (11.3, 15.0)	21.1 (18.5, 23.7)	35.4 (28.7, 42.2)	< 0.001
Myocardial infarction	2.3 (1.8, 2.8)	1.5 (0.9, 2.1)	3.6 (2.5, 4.6)	1.3 (0.2, 2.5)	0.002
Stroke	2.0 (1.5, 2.5)	1.8 (1.2, 2.5)	2.1 (1.3, 2.8)	2.8 (1.0, 4.5)	0.824

Patients without severe morbidities	Overall N = 639	NYHA I N = 283	NYHA II N = 267	NYHA III or IV N = 89	*P*
Any death	10.8 (9.7, 12.0)	7.0 (5.8, 8.2)	13.0 (11.0, 14.9)	23.2 (17.8, 28.5)	< 0.001
Heart failure hospitalization	6.2 (5.4, 7.1)	4.4 (3.3, 5.4)	7.2 (5.7, 8.8)	13.6 (9.1, 18.1)	0.018
Any death or heart failure hospitalization	14.3 (13.0, 15.7)	9.8 (8.2, 11.3)	16.9 (14.6, 19.3)	30.7 (24.0, 37.5)	< 0.001
Progression to severe aortic stenosis	3.9 (3.0, 4.7)	4.4 (3.2, 5.6)	3.3 (2.1, 4.5)	3.2 (0.6, 5.7)	0.012
AVR	2.5 (1.9, 3.0)	3.1 (2.2, 3.9)	1.9 (1.1, 2.6)	2.0 (0.4, 3.6)	0.003
Any death, heart failure hospitalization, or AVR	16.4 (14.9, 17.9)	12.1 (10.4, 13.9)	18.5 (15.9, 21.0)	33.1 (25.9, 40.3)	< 0.001
Myocardial infarction	2.2 (1.7, 2.7)	1.4 (0.9, 2.0)	3.5 (2.5, 4.6)	1.3 (0.03, 2.6)	0.002
Stroke	2.0 (1.5, 2.5)	1.9 (1.2, 2.6)	1.9 (1.1, 2.6)	3.0 (1.0, 5.0)	0.657

Rates are reported as events per 100 person-year follow-up with 95 % confidence intervals. *Overall, 517 patients (71 %) had echocardiography in follow-up. *P* value was calculated using the chi-square test or Fisher’s exact test. AVR, aortic valve replacement; NYHA, New York Heart Association

**Fig. 2:**
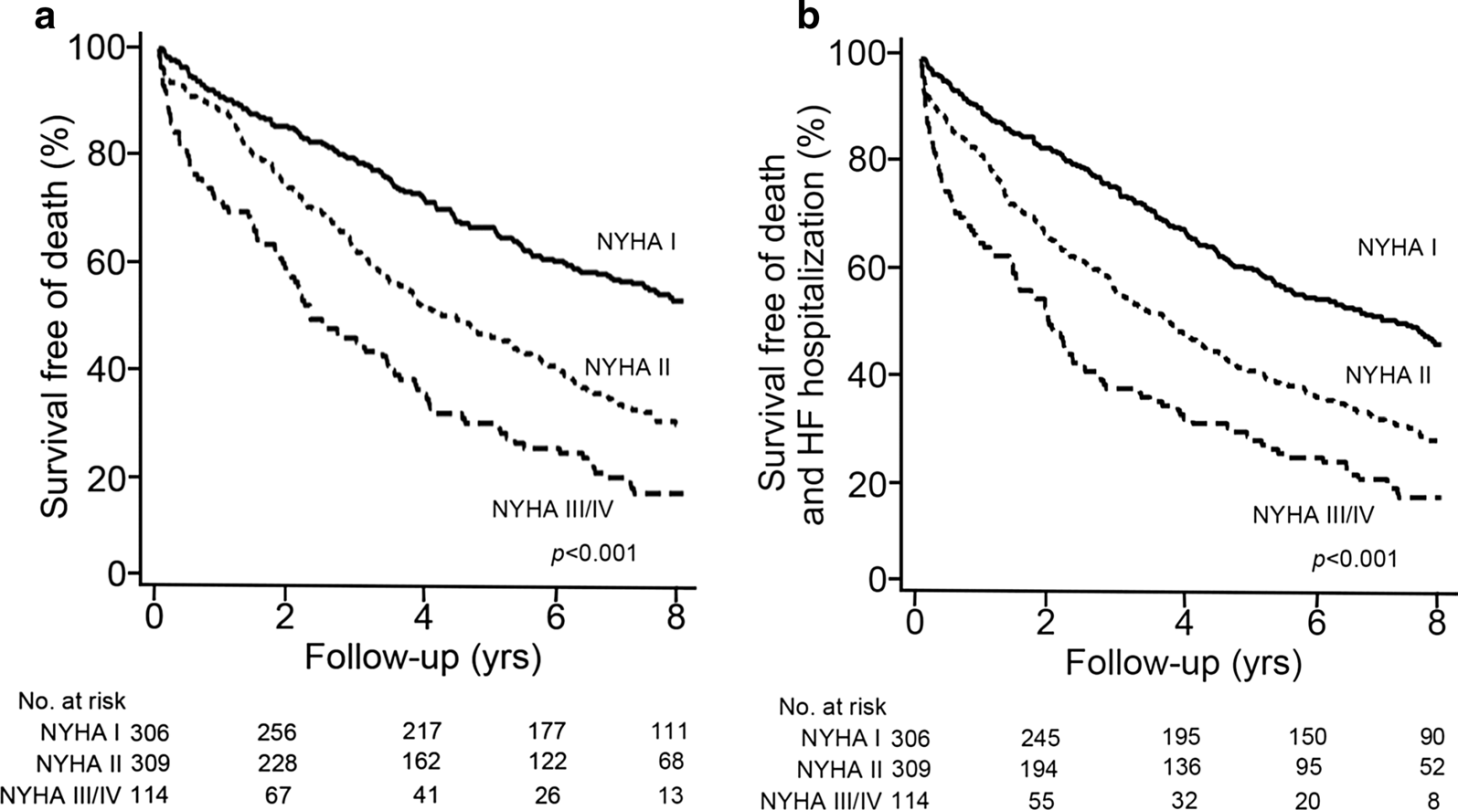
Survival according to baseline New York Heart Association (NYHA) functional class for the entire cohort. **a** Survival free of death. **b** Survival free of death and heart failure (HF) hospitalization

Worse NYHA class was related to higher rates of any death, HF hospitalization and the combined endpoint of death or HF hospitalization (all *p* < 0.05) (Table 3). Notably, among 306 patients with minimal or no symptoms (i.e., NYHA class I), the 5-year survival was 66.1 % (95 % CI: 60.8 % to 71.4 %), with an incidence of 7.9 (95 % CI: 6.6 to 9.2) per 100 person-year (Fig. 2; Table 3). For the combined endpoint of death or HF hospitalization, the 5-year survival for the NYHA class I patients was 57.1 % (95 % CI: 51.4–62.8 %), with an incidence of 10.7 (95 % CI: 9.2 to 12.3) per 100 person-year (Fig. 2; Table 3).

The trends in the rates of death and the combined endpoint of death or HF hospitalization were similar when patients with potential futility markers were excluded (all *p* < 0.001) (Table 3). Among the 639 patients without potential futility markers, the survival was 57.4 % (95 % CI: 53.5–61.3 %) at 5 years, and was 47.8 % (95 % CI: 43.9–51.7 %) at 5 years for the combined endpoint of death or HF hospitalization. Of note, for patients who had minimal or no symptoms (NYHA I) and who also were without potential futility markers (n = 283), the 5-year overall survival and survival free of the combined endpoint of death or HF hospitalization were 70.5 % (95 % CI: 65.2–75.8 %) and 60.7 % (95 % CI: 55.0–66.4 %), respectively. In addition, the 5-year overall survival was 58.2 % (95 % CI: 53.9–62.5 %) among 522 patients with preserved LV systolic function (≥50 %), while in whom with NYHA I, the survival was 67.6 % (95 % CI: 62.1–73.1 %) at 5 years. Moreover, this survivorship was similar when analyses were restricted to patients with minimal or no symptoms, without potential futility markers, and with preserved LV ejection fraction. In these patients (n = 262), the 5-year survival was 71.9 % (95 % CI: 66.4–77.4 %) with a death rate of 6.8 (95 % CI: 5.5 to 8.0) per 100 person-year.

## Progression to AVR and censoring

**Fig. 3:**
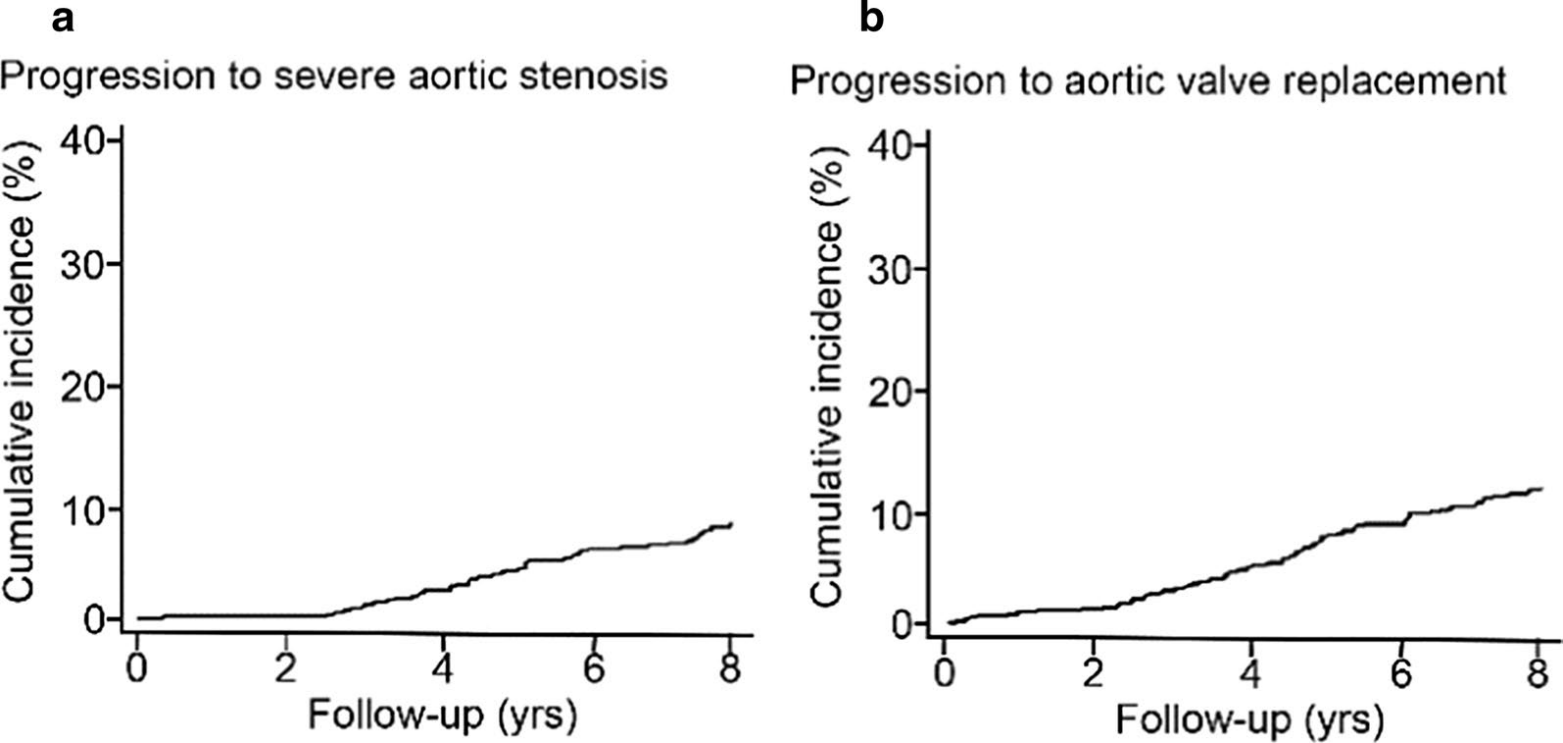
Disease progression for patients with moderate aortic stenosis.** a** Incidence of disease progression to severe aortic stenosis among patients with echocardiography in follow-up (n = 517). **b** Incidence of aortic valve replacement in the entire cohort (n = 729)

Echocardiographic follow-up was available in 517 patients (71 % of the cohort), at a median interval of 4.3 years (IQR: 2.3 to 6.8 years). Among them, 89 of these patients (17.2 %) had progression to severe AS, with an incidence of 3.9 (95 % CI: 3.1 to 4.7) per 100 person-year (Fig. 3; Table 3). Overall, AVR occurred in 83 patients (transcatheter, 55.4 %; surgical, 44.6 %), with an incidence of 2.5 (95 % CI: 1.9, 3.0) per 100 person-year (Fig. 3; Table 3). The median time to AVR for these patients was 4.4 years (IQR: 1.9 to 7.9 years).

**Fig. 4:**
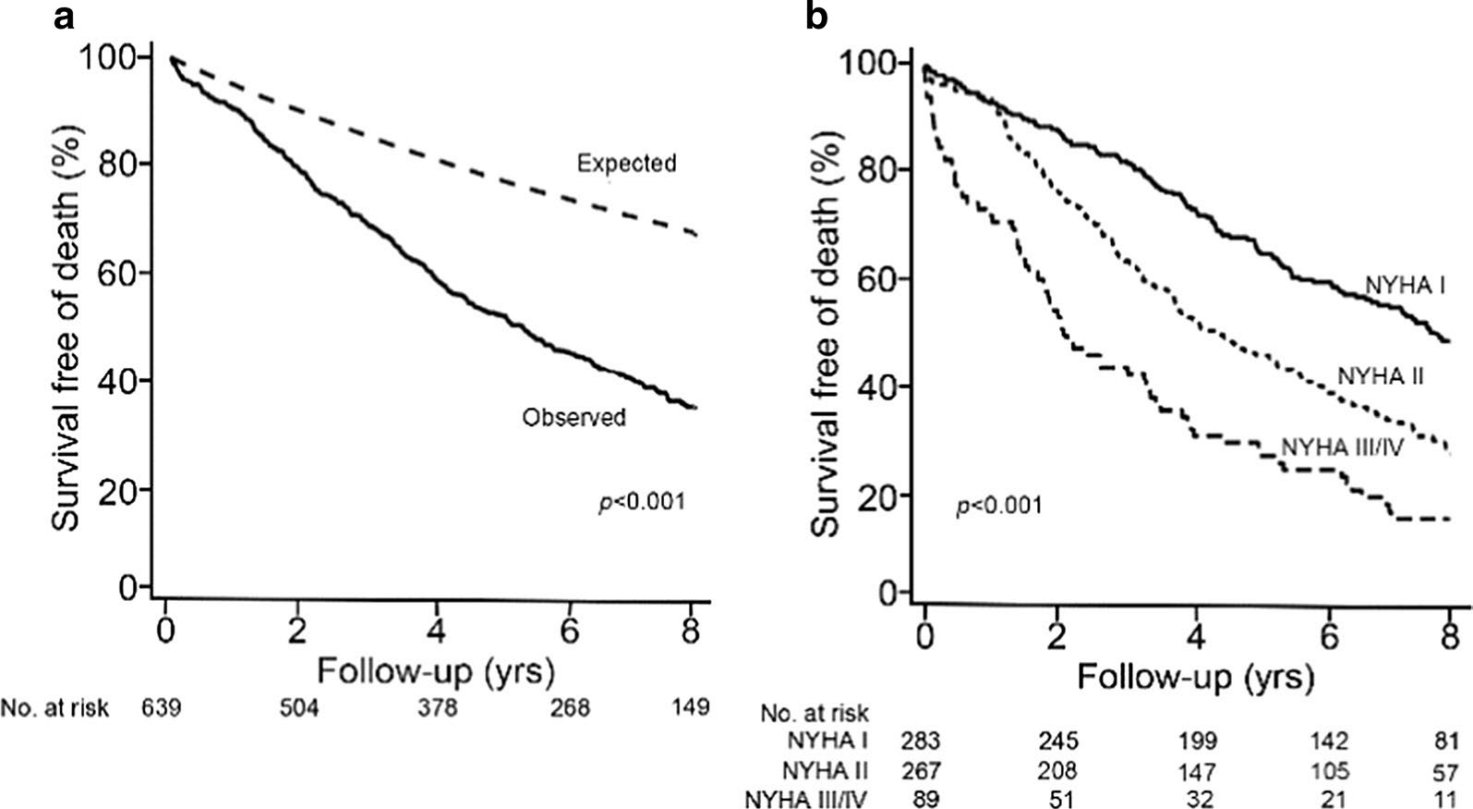
Survival in patients with moderate aortic stenosis who were without severe morbidities, with survival times censored at aortic valve replacement (N = 639).** a** Observed survival (solid) in comparison to the expected survival based on the age- and sex-matched total Minnesota population (dashed). **b** Observed survival according to New York Heart Association (NYHA) functional class

Survivorship analyses were similar with and without censoring for occurrence of AVR in follow-up. Overall, the 5-year survival and survival free of the combined endpoint of death or HF hospitalization were 52.7 % (95 % CI: 49.0 % to 56.4 %) and 43.5 % (95 % CI: 39.8 % to 47.2 %) with censoring for AVR, respectively. For the patients who had minimal or no symptoms (i.e., NYHA class I), these estimates were 66.4 % (95 % CI: 60.9 % to 71.9 %) and 57.3 % (95 % CI: 51.6 % to 63.0 %), respectively. Similarly, the survivorship was unchanged in a subset of patients without severe co-morbidities and with censoring for occurrence of AVR, with a 5-year survival of 58.0 % (95 % CI: 54.1 % to 61.9 %) (Fig. 4). For patients without futility markers and who had minimal or no symptoms, the estimate survival with censoring for AVR was 70.8 % (95 % CI: 65.3 % to 76.3 %) (Fig. 4). Moreover, for patients with minimal or no symptoms, no potential futility markers, and preserved LV function, the 5-year survival with censoring for AVR was 72.0 % (95 % CI: 66.3–77.7 %) (Additional file 1: Figure 2). For the combined endpoint of death or HF hospitalization, this survival was 61.7 % (95 % CI: 55.6–67.8 %).

## Multivariate models

In univariate and survival analyses, traditional prognostic echocardiographic parameters (e.g., LV ejection fraction < 50 %, stroke volume index < 35 ml/m^2^, and rapid AS progression rate [Vmax > 0.3 m/s/y]) were associated with worse outcomes in this cohort of patients (Additional file 1: Figures 3–5). In multivariate models, age, NYHA functional class, low LV ejection fraction and high peak aortic velocity were associated with all-cause mortality with AVR modeled as a time-dependent variable (Table 4). These associations were present in analyses of the overall population, as well as in analyses that excluded patients with potential futility markers (Table 4). These same variables were also independently associated with the occurrence of the composite endpoint of death or HF hospitalization in patients with or without potential futility markers (Table 4).

**Table 4:** Multivariate models. Cox proportional hazard regression analyses for predicting outcomes are shown

Variables	All patients			Patients without severe morbidities								
	Any death			Any death/HF hospitalization			Any death			Any death/HF hospitalization		
	HR	95 % CI	*p* value	HR	95 % CI	*p* value	HR	95 % CI	*p* value	HR	95 % CI	*p* value
Age (per 1-year)	1.04	1.03–1.05	< 0.001	1.03	1.02–1.05	< 0.001	1.05	1.04–1.06	< 0.001	1.04	1.03–1.05	< 0.001
BSA (per 1 kg/m^2^)	0.50	0.29–0.85	0.010	0.60	0.37–0.98	0.041	–	–	–	–	–	–
Occurrence of AVR*	0.77	0.49–1.19	0.237	1.00	0.63–1.57	0.986	0.86	0.54–1.36	0.510	0.89	0.55–1.45	0.643
NYHA II	1.39	1.09–1.76	0.007	1.37	1.10–1.70	0.005	1.37	1.06–1.78	0.016	1.29	1.02–1.64	0.034
NYHA III or IV	1.67	1.20–2.31	0.002	1.38	1.01–1.90	0.045	1.72	1.20–2.47	0.003	1.45	1.02–2.04	0.036
Stroke/TIA	1.44	1.14–1.83	0.002	1.32	1.05–1.66	0.016	1.58	1.22–2.03	< 0.001	1.37	1.07–1.75	0.012
Hypertension	–	–	–	1.41	1.10–1.82	0.007	–	–	–	1.35	1.03–1.78	0.031
Diabetes	1.25	1.01–1.54	0.039	1.24	1.02–1.52	0.030	–	–	–	1.26	1.01–1.57	0.040
Chronic lung disease	1.53	1.22–1.91	< 0.001	1.35	1.09–1.68	0.007	1.52	1.17–1.96	0.002	1.44	1.12–1.85	0.004
CKD stage ≥ III	1.43	1.16–1.77	0.001	1.24	1.00-1.53	0.046	1.41	1.12–1.78	0.003	1.33	1.06–1.67	0.014
Anemia	1.60	1.30–1.98	< 0.001	1.50	1.23–1.83	< 0.001	1.64	1.32–2.04	< 0.001	1.45	1.18–1.79	0.001
Liver cirrhosis	3.36	1.75–6.45	< 0.001	2.70	1.41–5.15	0.003	3.48	1.68–7.22	< 0.001	2.22	1.07–4.59	0.032
Dementia	1.85	1.31–2.61	< 0.001	1.77	1.27–2.46	0.001	1.98	1.33–2.95	< 0.001	1.59	1.07–2.35	0.022
Malignancy	11.75	6.89–20.06	< 0.001	7.57	4.50-12.73	< 0.001	–	–	–	–	–	–
Excessive frailty	2.38	1.51–3.76	< 0.001	2.90	1.85–4.56	< 0.001	–	–	–	–	–	–
LV ejection fraction (per 1 %)	0.98	0.98–0.99	0.001	0.98	0.97–0.99	< 0.001	0.98	0.97–0.99	0.002	0.97	0.97–0.98	< 0.001
Peak aortic velocity (per 1 m/s)	1.37	1.13–1.67	0.002	1.37	1.14–1.64	0.001	1.34	1.08–1.67	0.008	1.39	1.14–1.70	0.001
≥ moderate TR	1.47	1.13–1.92	0.005	1.32	1.01–1.72	0.041	1.47	1.09–1.98	0.012	1.43	1.08–1.91	0.014
≥ moderate RV dysfunction	2.58	1.61–4.16	< 0.001	2.03	1.27–3.24	0.003	2.59	1.46–4.60	0.001	1.73	1.00–2.99	0.051

*AVR as a time-dependent variable. AVR, aortic valve replacement; BSA, body mass index; CI, confidence interval; CKD, chronic kidney disease; HF, heart failure; HR, hazard ratio; LV, left ventricular; NYHA, New York Heart Association; RV, right ventricular; SV, stroke volume; TIA, transient ischemic attack; TR, tricuspid regurgitation

## Discussion

The present investigation is a real-world examination of outcomes in patients with moderate AS evaluated in a large health system that includes outpatient care clinics and tertiary referral centers. The principal findings are: (1) Patients with moderate AS can have impaired prognosis, with a 5-year survival only 52 % observed in this study; (2) The heightened risk of death remains even when focusing on patients with minimal or no symptoms, no LV systolic dysfunction and no potential futility markers, in whom the 5-year survival was only 72 %; (3) These outcomes were observed while there were low rates of progression to severe AS (∼4 % per year) and need for AVR (∼2.5 % per year), and after accounting for the occurrence of such events with censoring; (4) Advanced age, worse NYHA functional class, low ejection fraction, high peak aortic velocity and severe comorbidities were associated with worse outcomes. Taken together, these findings highlight the prognostic impact of AS, even when only moderate in severity.

While there has been intense focus on the evaluation and management of patients with severe AS, those with moderate disease may be vulnerable with an impaired prognosis. The present investigation was undertaken as a real-world, longitudinal study of 729 patients with moderate AS (median AVA, 1.5 cm^2^) observed over a median follow-up of 5.0 years. In our study, the 5-year survival was 52 % for the entire cohort. Hospitalization for HF occurred at rate of ~ 6.0 % per year. Certainly, this survivorship is better than what would be expected for severe, symptomatic aortic stenosis, where the mortality rates are commonly ~ 25 % per year or more [7]. Nevertheless, the annual mortality rate of 12.6 % in our study is notable, and markedly worse than expected. Our findings mirror the results of Strange et al. [7], who utilized a large national echocardiography database in Australia and reported a 5-year mortality rate of 56 % for 3,315 patients with moderate AS. Similarly, in a separate study of 508 moderate AS patients by Delesalle et al., the 6-year survival was only 53 % [8]. Taken together, the risk of death in patients with moderate AS is remarkable, and deserves further scrutiny regarding potential management to improve their prognosis.

Importantly, our findings extend the results of other studies, with a more granular analysis of the influence of symptoms, LV systolic function, occurrence of AVR, and morbidities on survival of these patients. Not surprisingly, worse functional class was strongly associated with the presence of morbidities and poor survival, with an annual mortality rate of 25 % for those with most impairment (i.e., NYHA III/IV), and many morbidities were independently predictive of the long-term outcomes. Nonetheless, for those patients with minimal or no symptoms and with no evidence of futility, the 5-year survival was still only 71 % with an annualized death rate of ∼7.0 % per year. This survivorship also was similar when we restricted analyses to those patients with minimal impairment, who had preserved LV function and no futility markers, with and without censoring for occurrence of AVR (i.e., 5-year survival free of all-cause mortality, ~ 72 %).

While a study of a younger population of patients with moderate AS (n = 514) by Lancelotti et al. [4] found a 4-year survival of 89 %, it is recognized that AS, even when not severe, can impart significant myocardial architectural changes. Increases in LV mass, mid-wall fibrosis, and extracellular volume can occur with progression of AS, even when not severe [18–22]. As reversibility of cardiac damage with AVR has been shown in severe AS, there is rationale for consideration of AVR in patients with moderate AS and LV dysfunction [23–26]. Whether such therapy should be extended to all patients with moderate AS, with or without preserved LV function, may be a consideration. The high mortality rate in our study, as has been observed in others, invites scrutiny into the possible benefit of AVR in patients with preserved LV function and with minimal clinical impairment, especially given that procedural mortality with modern transcatheter or surgical techniques is now relatively low, and often < 1–3 % [27]. Certainly, a survival benefit of such therapy for patients with moderate AS would only be hypothetical until tested in prospective, randomized clinical trials [28].

The present study was a longitudinal, yet retrospective analysis of patients from hospitals and outpatient clinics, therefore subject to selection bias. Nonetheless, our findings suggest the need for close clinical follow-up and better clinical management in patients with moderate AS, who are at significant risk of death, even when minimally or asymptomatic. A single positive echocardiographic parameter for defining moderate AS also posed limitations. In addition, in the cohort of patients with no symptoms, no severe morbidities, and preserved LV function, some patients with a prior history of myocardial infarction were included and that morbidity could have impacted their survival. Lastly, we were limited in our ability to adjudicate cardiovascular from non-cardiovascular deaths, which would be important to determine a potential protective role for AVR.

## Conclusions

Our study demonstrates significant mortality rates for patients with moderate AS in the setting of hospital and outpatient clinic, and thus highlights the need to reexamine thresholds for clinical surveillance and intervention in patients with moderate AS.

## Supplementary Information

**Addititional file 1.** **Figure 1** Study flow-chart. AVA, aortic valve area; AS, aortic stenosis; F/U, follow up. **Figure 2** Survival of patients with moderate aortic stenosis who were without severe morbidities or left ventricular dysfunction, with survival times censored at aortic valve replacement (N = 461). (A) Observed survival in comparison to the expected survival based on the age- and sex-matched total Minnesota population. (B) Observed survival according to New York Heart Association (NYHA) functional class. **Figure 3.** Survival of patients with moderate aortic stenosis according to stroke volume index (SVi) < 35 ml/m2 vs. ≥ 35 ml/m2. (A) Observed survival free of death. (B) Observed survival free of death or heart failure (HF) hospitalization. **Figure 4.** Survival of patients with moderate aortic stenosis according to left ventricular ejection fraction (EF) < 50 % vs. ≥ 50 %. (A) Observed survival free of death. (B) Observed survival free of death or heart failure (HF) hospitalization. **Figure 5.** Survival of patients with moderate aortic stenosis according to rate of aortic stenosis progression (Vmax > 0.3 m/s/y vs. ≤ 0.3 m/s/y) in patients with available echocardiographic follow-up (N = 505). (A) Observed survival free of death. (B) Observed survival free of death or heart failure (HF) hospitalization.

## Data Availability

The datasets used and/or analysed during the current study are available from the corresponding author on reasonable request.

## Abbreviations

AS: aortic stenosis
AVR: aortic valve replacement
AVA: aortic valve area
CIs: confidence intervals
DI: dimensionless index
HF: heart failure
IQR: interquartile range
LV: left ventricular
MI: myocardial infarction
NYHA: New York Heart Association
VTI: velocity time integral
