# Supplementary material for: Natural history observations in moderate aortic stenosis
Source: BMC Cardiovasc Disord. 2021 Feb 19;21:108. doi: 10.1186/s12872-021-01901-1 (PMC7893941; doi:10.1186/s12872-021-01901-1)

**Natural History Observations in Moderate Aortic Stenosis**

**Additional file 1**

Yu Du, Mario Gössl, Santiago Garcia, Maurice Enriquez-Sarano, Joao L. Cavalcante, Richard Bae, Go Hashimoto, Miho Fukui, Bernardo Lopes, Aisha Ahmed, Christian Schmidt, Larissa Stanberry, Ross Garberich, Steven M. Bradley, Robert Steffen, Paul Sorajja

**Supplement 1.** Study flow-chart. AVA, aortic valve area; AS, aortic stenosis; F/U, follow up.

**Supplement 2.** Survival of patients with moderate aortic stenosis who were without severe morbidities or left ventricular dysfunction, with survival times censored at aortic valve replacement (N=461). (A) Observed survival in comparison to the expected survival based on the age- and sex-matched total Minnesota population. (B) Observed survival according to New York Heart Association (NYHA) functional class.

**Supplement 3.** Survival of patients with moderate aortic stenosis according to stroke volume index (SVi) <35 ml/m^2^ vs ≥35 ml/m^2^. (A) Observed survival free of death. (B) Observed survival free of death or heart failure (HF) hospitalization.

**Supplement 4.** Survival of patients with moderate aortic stenosis according to left ventricular ejection fraction (EF) <50% vs ≥50%. (A) Observed survival free of death. (B) Observed survival free of death or heart failure (HF) hospitalization.

**Supplement 5.** Survival of patients with moderate aortic stenosis according to rate of aortic stenosis progression (Vmax > 0.3 m/s/y vs ≤ 0.3 m/s/y) in patients with available echocardiographic follow-up (N=505). (A) Observed survival free of death. (B) Observed survival free of death or heart failure (HF) hospitalization.

**Supplement 1**

**
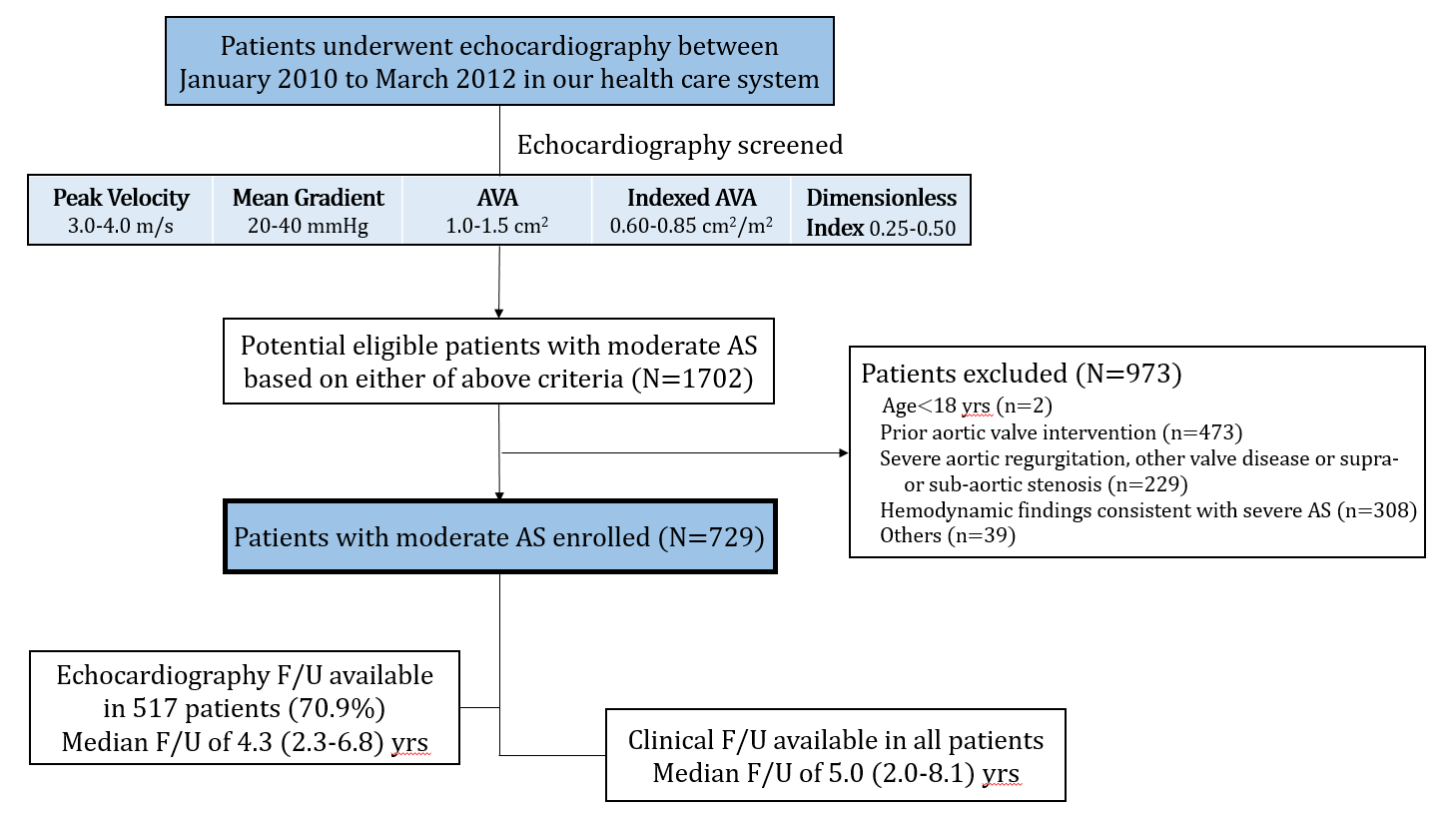
**

**Supplement 2**


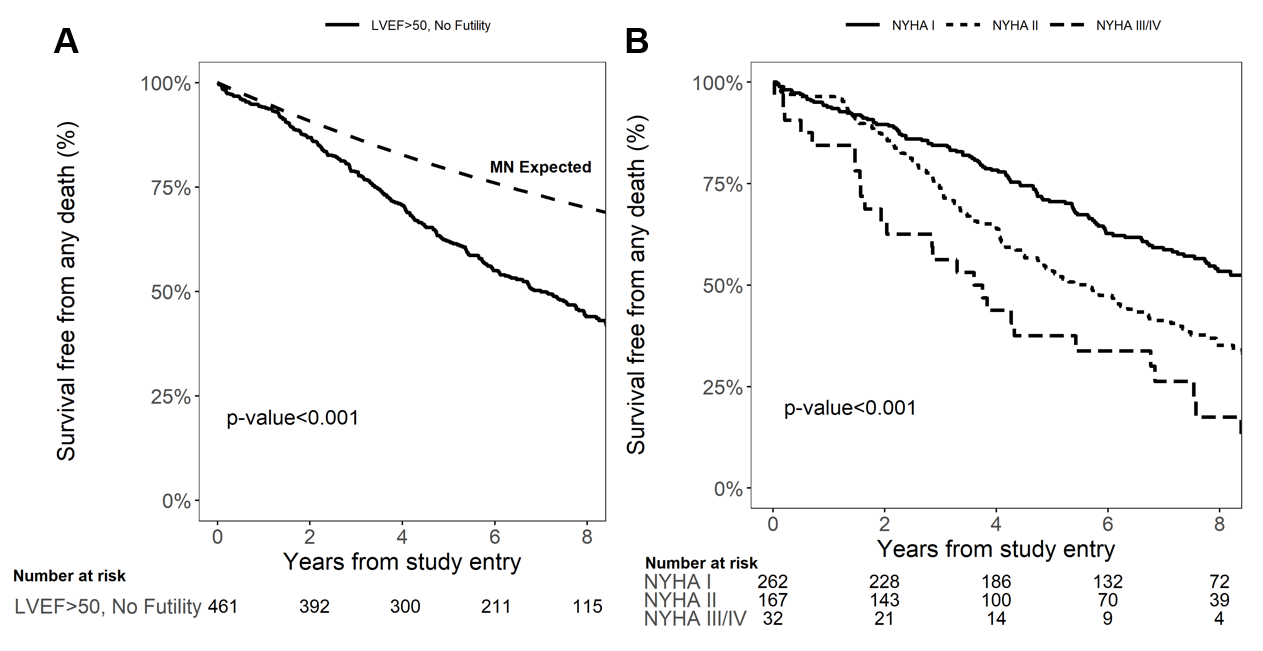


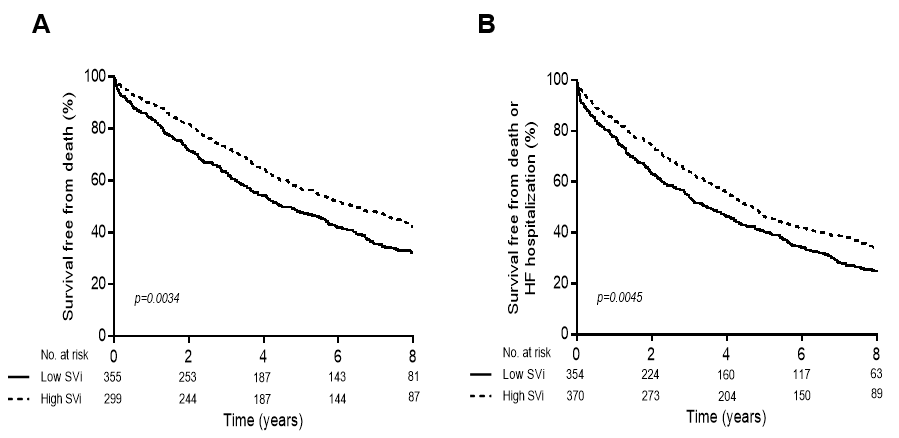


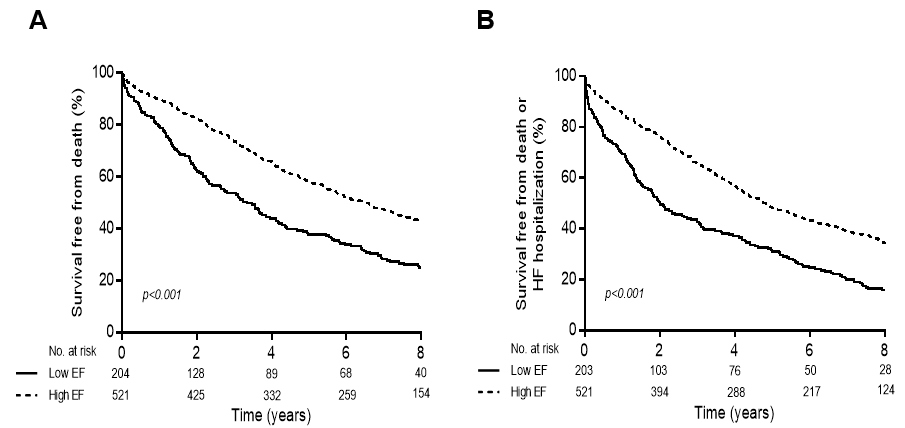


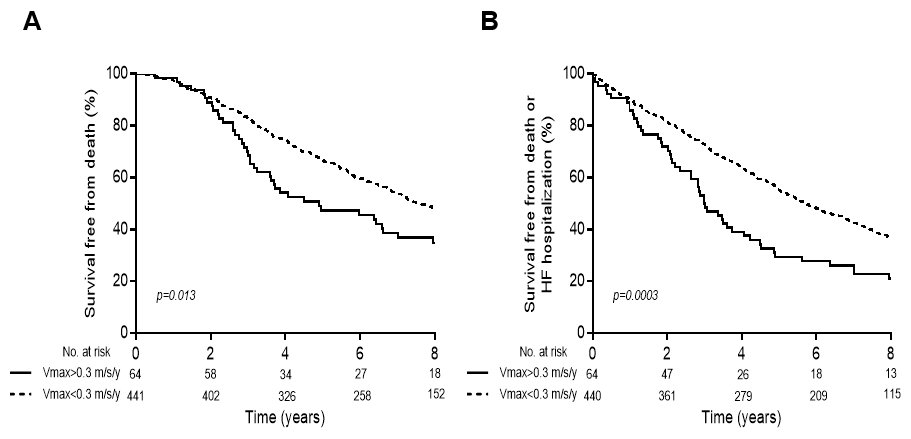

Supplement: Supplementary file 1 — Addititional file 1. Figure 1 Study flow-chart. AVA, aortic valve area; AS, aortic stenosis; F/U, follow up. Figure 2 Survival of patients with moderate aortic stenosis who were without severe morbidities or left ventricular dysfunction, with survival times censored at aortic valve replacement (N = 461). (A) Observed survival in comparison to the expected survival based on the age- and sex-matched total Minnesota population. (B) Observed survival according to New York Heart Association (NYHA) functional class. Figure 3. Survival of patients with moderate aortic stenosis according to stroke volume index (SVi) < 35 ml/m2 vs. ≥ 35 ml/m2. (A) Observed survival free of death. (B) Observed survival free of death or heart failure (HF) hospitalization. Figure 4. Survival of patients with moderate aortic stenosis according to left ventricular ejection fraction (EF) < 50 % vs. ≥ 50 %. (A) Observed survival free of death. (B) Observed survival free of death or heart failure (HF) hospitalization. Figure 5. Survival of patients with moderate aortic stenosis according to rate of aortic stenosis progression (Vmax > 0.3 m/s/y vs. ≤ 0.3 m/s/y) in patients with available echocardiographic follow-up (N = 505). (A) Observed survival free of death. (B) Observed survival free of death or heart failure (HF) hospitalization. [file 12872_2021_1901_MOESM1_ESM.docx]
